# Clinical Effectiveness of Dry Needling on Myofascial Trigger Points in Horses: A Prospective Algometric Controlled Study

**DOI:** 10.3390/ani15152207

**Published:** 2025-07-27

**Authors:** Maria Calatayud-Bonilla, Jorge U. Carmona, Marta Prades

**Affiliations:** 1 Department of Animal Medicine and Surgery, Universitat Autònoma de Barcelona (UAB), 08193 Barcelona, Spain; marta.prades@uab.cat; 2 Grupo de Investigación Terapia Regenerativa, Departmento de Salud Animal, Universidad de Caldas, 170004 Manizales, Colombia; carmona@ucaldas.edu.co

**Keywords:** horse, trigger point, pain, palpation, algometry, dry needling, myofascial pain syndrome, equine brachiocephalic muscle

## Abstract

Myofascial pain syndrome (MPS) is caused by trigger points (TrPs): hypersensitive spots in taut muscle bands that impair function and cause pain. Dry needling (DN) is a common treatment in humans, but evidence in horses is limited. This prospective, controlled study evaluated the effectiveness of DN in reducing TrP-related pain in the brachiocephalic muscle of horses. Of the 98 horses enrolled, 66 were allocated to a treatment group receiving weekly DN sessions for three weeks, while 32 were assigned to a control group with no intervention. Pain and function were assessed using pressure algometry, a numerical rating scale (NRS), a functional total test score (FTTS), and behavioral indicators including jump sign (JS), equine pain face (EPF), and local twitch responses (LTRs). Assessments were performed at baseline and at 0, 4, 24, and 72 h post-intervention. Results indicate a significant increase in pressure pain thresholds (*p* < 0.001), especially after the second and third sessions. Both NRS and FTTS improved significantly over time (*p* < 0.001), and LTRs progressively decreased. EPF and JS showed minimal variation. These results support the use of DN to reduce local muscle pain and improve function in horses with TrPs. Further robust studies are warranted to refine protocols and investigate long-term effects.

## Simple Summary

Muscle pain can impact how animals feel, move, and perform. A common type of pain, known as myofascial pain, is associated with trigger points: small, sensitive spots in muscle that may limit motion and cause discomfort. In human medicine, a technique called dry needling, where a thin, sterile needle is inserted into these trigger points, has shown good results in reducing pain. This study explores whether dry needling could help horses with muscle pain in the neck area. We treated horses once a week for three weeks and compared them to untreated horses. The treated group showed reduced pain sensitivity and improved muscle function, while untreated horses showed no change. These findings suggest that dry needling may be an effective non-pharmacological option for managing muscle pain in horses. Improving how we detect and treat this type of pain could help enhance horses’ comfort, movement, and overall well-being.

## 1. Introduction

Myofascial pain syndrome (MPS) is characterized by the presence of myofascial trigger points (TrPs), which produce a constellation of sensory, motor, and autonomic symptoms [1,2,3]. A TrP is defined as a hyperirritable spot within a taut band (TB) of skeletal muscle. It may provoke local pain, pain during contraction or stretching, muscle weakness, motor disturbances, vasovagal signs, autonomic manifestations, and referred pain [4,5].

The TB, a hallmark of TrPs, is a hardened and palpable band of muscle fibers that contracts upon stimulation. This may trigger a LTR, an involuntary contraction linked to a spinal reflex and motor endplate dysfunction [1]. In human patients, TrPs are frequently diagnosed in musculoskeletal pain syndromes, with a global lifetime prevalence of up to 85% [6,7,8,9]. In veterinary medicine, awareness of their clinical relevance has grown [10,11,12], and prevalence data are emerging for equine populations [13].

Current pathophysiological research suggests that acute muscle overload or repetitive activity can lead to TrP formation, likely due to muscle fatigue and increased acetylcholine release at the motor endplate, elevated cytosolic calcium levels, or both [7,14]. These findings support the integrated hypothesis [4,15,16]. Additional studies highlight the role of calcium and the sympathetic nervous system in TB maintenance [17] and emphasize the importance of spontaneous electrical activity (SEA) [18], a marker of excess acetylcholine release from dysfunctional endplates [19]. SEA has been detected in horses, especially in the cleidobrachial portion of the brachiocephalicus muscle (BM) [11], reinforcing the cross-species consistency of TrP physiology among humans, rabbits, and equines [11,20].

The diagnosis of TrPs is based on clinical criteria, including the presence of a TB, a hypersensitive tender spot within the band, and pain reported by the patient [5]. Although no universally accepted diagnostic standards exist for equine TrPs, foundational studies suggest that human-based principles may be applicable to horses [10,11,21].

Accurate TrP identification requires clinical expertise and palpation skills [22,23]. Previous studies have demonstrated high inter-rater reliability in TrP detection [23,24,25,26]. In horses, TrPs are linked to clinical conditions such as back pain and girth aversion, and share several features with human TrPs, including TBs, LTRs, and pain upon palpation. Electromyographic patterns consistent with TrPs have also been reported in equines [10,11,27]. Recently, the impact of dry needling (DN) on equine biomechanics has been explored using AI-assisted gait analysis [28].

Pain assessment in horses presents a unique challenge, as prey animals often conceal pain as a survival strategy [29]. In animals, pain is defined as a sensory experience resulting from tissue damage that elicits protective and behavioral responses [30]. Observable indicators include postural changes, gait alterations, facial expressions such as the equine pain face (EPF) [31], avoidance behaviors, a jump sign (JS), and sudden withdrawal responses upon palpation [32,33,34]. Simpler tools, including the numerical rating scale (NRS), visual analog scale (VAS), and simple descriptive scale, are also employed, with the NRS considered the most reliable in equine clinical practice [32,35].

Manual palpation is used to detect pain sensitivity via behavioral responses to TrP stimulation. Pressure pain thresholds (PPTs), in contrast, offer an objective and quantifiable measure of pain sensitivity. Although pain is inherently subjective [36], sensitivity refers to the measurable response to a noxious stimulus [37]. Both palpation and gait evaluation remain fundamental components of equine physical rehabilitation [38].

Dry needling is a therapeutic intervention involving the insertion of fine, sterile needles into TrPs to deactivate them. DN has been shown to reduce local pain and improve muscle function [1]. However, a known side effect is post-needling soreness (PNS), characterized by transient, localized discomfort and stiffness. The severity of PNS may correlate with the number of LTRs elicited during treatment [39,40].

This study aims to determine whether DN can reduce TrP-induced local pain in horses, specifically in the distal portion of the BM. Building on previous findings [41], we evaluate the effects of DN on TrP-associated pain in a large cohort of horses. Additionally, we explore the relationship between the number of LTRs elicited and the evolution of local pain, JS, and PNS. To assess the functional impact, owners completed a functional total test score (FTTS) and a NRS to monitor changes in pain perception.

## 2. Materials and Methods

### 2.1. Ethical Considerations and Study Population

Although this study involved invasive physiotherapy procedures, it was conducted under routine clinical conditions, and therefore did not require approval from the Animal Experimentation Ethics Committee, in accordance with applicable institutional and national guidelines. Nevertheless, informed consent was formally obtained from horse owners through the Human Experimentation Ethics Committee of the Universitat Autònoma de Barcelona (UAB) under procedure identifier 5618.

The study population consisted of 98 horses aged between 5 and 15 years, housed in stalls, paddocks, or a combination of both housing systems. Horses were recruited through an online call for participation: a short video describing the study was posted by M.C.-B. on social media on 19 October 2021. All study procedures were conducted at three equestrian centers (facilities with individual paddocks within a pine forest; facilities with combined stalls and paddocks; and facilities with stalls with partial access to paddocks).

Horses were randomly allocated to one of two groups using a simple randomization process based on a computer-generated list of random numbers: a treatment group (TG; n = 66) or a control group (CG; n = 32). The TG included 34 competition horses, 19 school horses, and 13 inactive horses, whereas the CG comprised 22 competition horses and 10 inactive horses.

To qualify for inclusion, horses were required to exhibit a TrP in the distal portion of the BM, diagnosed according to established equine physiotherapy criteria, which included static and dynamic postural evaluation, palpation for muscle tone and reactivity, joint range of motion, gait analysis, and compensatory pattern assessment. This involved identification of a TB, the presence of a hypersensitive spot, and a clear pain response upon palpation, defined as a nocifensive withdrawal reaction such as sudden movement away from the examiner, weight shift, skin twitching, or muscle fasciculation [42]. Palpation pressure was manually applied and progressively increased until a reaction was observed. No device was used to standardize force. Only the BM was examined for trigger points; epaxial or other regions were not assessed. Additional inclusion criteria required that horses be clinically healthy (i.e., with no diagnosed musculoskeletal conditions and no lameness reported or observed at enrollment), and have current veterinary records and vaccinations.

Exclusion criteria were defined to ensure animal welfare and scientific rigor. Horses exhibiting aggressive or inappropriate behavior, needle aversion, or poor handling tolerance were excluded.

### 2.2. Study Design and Interventions

This prospective, controlled intervention study was conducted over a three-week period. Prior to enrollment, horse owners received detailed written and verbal information regarding the study objectives, procedures, pre-treatment requirements, and post-treatment care instructions. Additionally, owners provided written informed consent—approved by the Institutional Animal Ethics and Welfare Committee—which outlined the nature of the intervention, potential benefits, and associated risks.

Baseline data were recorded for each subject, including sex, age, body weight, behavioral characteristics, previous medical history, type of training, and housing conditions (stabled or pastured).

Prior to each intervention session, owners completed both the FTTS (for a complete description of this test, see Appendix A) and the NRS to evaluate their horse’s functional status and perceived pain levels. Notably, the NRS rated perceived pain on a scale from 0 (no pain) to 10 (maximum pain).

The entire operational phase of the study, including all interventions performed on the animals, was conducted by M.C.-B., an equine physiotherapist with over 20 years of clinical experience in myofascial pain management and TrP identification. The physiotherapist’s expertise ensured high intra-subject reliability for both manual palpation and TrP localization [24,25,26]. Four physiotherapy auxiliary students (PAS), trained in the study protocol, assisted with behavioral observations, data recording, and algometric measurements. To minimize measurement bias, the physiotherapist remained blinded to the algometry results throughout the study [43,44,45].

#### 2.2.1. Pre-Treatment and Post-Treatment Conditions

To ensure methodological consistency, pre-treatment and post-treatment conditions were standardized as follows: (1) Prior to each intervention, horses were permitted to undergo light training, provided that such activity was completed before the treatment session. (2) All horses were groomed immediately prior to the intervention to facilitate palpation and ensure skin cleanliness. (3) Interventions were performed in a quiet, low-stress environment, with horses tied using a lead rope that permitted limited but safe mobility.

For the first two days following each intervention, training was limited to maintaining a therapeutic balanced posture. Exercises involving lateral movements, small circles, extended trot, or dressage-type activities were strictly avoided. Normal training routines were resumed 72 h post-intervention. Furthermore, on days when follow-up measurements were scheduled, any training was conducted prior to data collection to minimize confounding effects on outcome variables. These post-treatment care guidelines were uniformly applied throughout the three-week study period.

#### 2.2.2. Intervention Sessions (First Day of Each Week)

At each weekly intervention session, following grooming, horses were positioned on a stable non-slip surface and were loosely tied in the designated treatment area. To standardize the procedure and minimize potential side bias, all interventions were consistently initiated on the left side.

Before commencing the intervention, the PAS assessed the horses’ facial expressions using an EPF scale. The evaluation lasted 15 s, during which specific facial action units were monitored, including ear position, eye shape, nostril dilation, and lip and chin tension. Each feature was scored as either 0 (absent) or 1 (present) if observed at least once during the assessment period. This simplified binary scoring system was adapted to enhance consistency and feasibility under field conditions, while remaining aligned with the validated EPF methodology.

Subsequently, a systematic manual palpation of the distal portion of the BM was conducted to identify TB and hypersensitive TrP, using the cranial angle of the scapula as a consistent anatomical landmark [42]. Inclusion in the study cohort required the presence of at least one TrP associated with a JS—a characteristic reflexive response indicative of nociceptive sensitivity. During palpation, the PAS monitored and recorded the presence of JS, scored as absent (0) or present (1), and were instructed to notify the physiotherapist immediately if excessive discomfort occurred.

Baseline algometry measurements were then taken. For the intervention, the TrP selected for dry needling was the one exhibiting the lowest PPT as determined by algometry, combined with a clearly observable nocifensive behavioral response, such as a JS, during manual palpation. This operational definition was applied to ensure objective and reproducible TrP selection.

After all assessments, DN was performed at the identified TrP site, with subsequent measurements taken immediately post-intervention (POST0) and four hours post-intervention (POST4 h).

#### 2.2.3. Follow-Up Measurements (Second and Fourth Days of Each Week)

**Figure 1:**
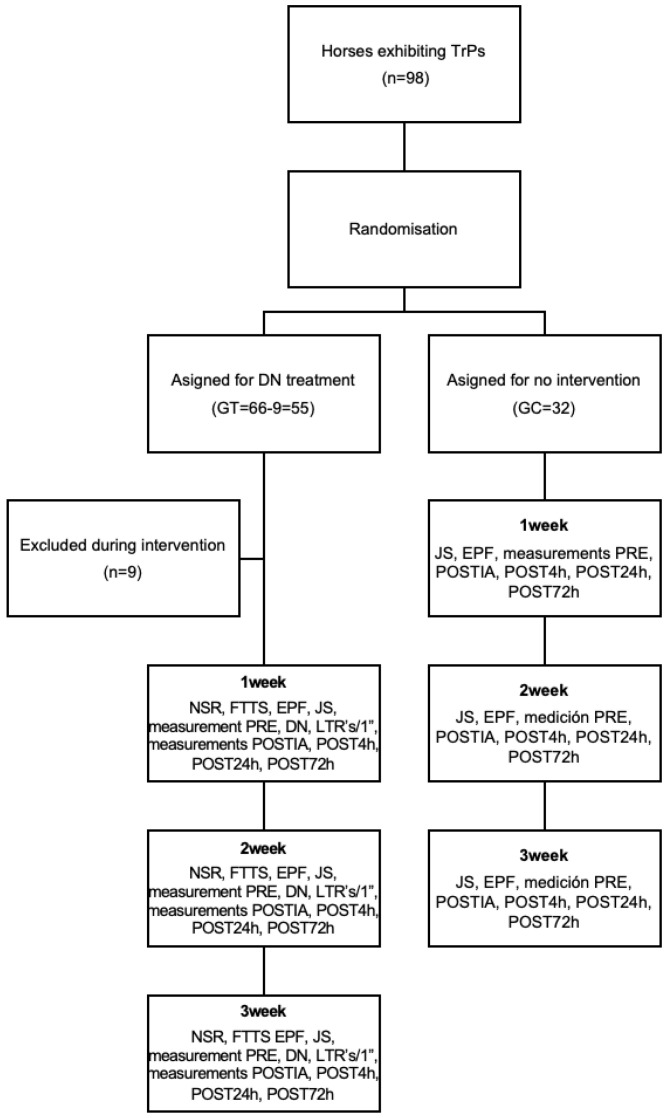
Design of the study. (TrPs: trigger points; DN: dry needling; NSR: numerical rate scale; FTTS: functional total test score; EPF: equine pain face; JS: Jump sign; LTRs: local twitch responses; POSTIA: immediately after; POST4 h: after 4 h; POST24 h: after24 h; POST72 h: after 72 h).

Follow-up assessments were conducted at 24 h (POST24 h) and 72 h (POST72 h) post-intervention. Horses were restrained in the same location and manner as during the intervention sessions to ensure consistency in the handling and environmental conditions. An overview of the study design is presented in Figure 1.

### 2.3. Algometry Protocol

All PPT measurements were performed by M.C.-B. at the identified TrP sites using a calibrated Force Dial™ FDK/FDN Series algometer (Wagner Instruments, Greenwich, MT, USA) fitted with a 1 cm^2^ rubber tip. The device was calibrated prior to each measurement session following the manufacturer’s standard protocol to ensure accuracy and consistency across all time points. The use of a small-diameter probe allowed for localized and consistent stimulation of nociceptors [46].

For each site, three consecutive measurements were obtained, with 4–5 s intervals between readings [45,46]. The algometer was applied perpendicularly to the skin over the TrP at a consistent rate until a clearly observable pain response (behavioral reaction or avoidance) was elicited. The TrP selected for intervention was the one exhibiting the lowest PPT, in combination with a clear nocifensive behavioral response such as a JS, ensuring objective and reproducible selection criteria.

All PPT values were recorded in kilogram-force per square centimeter (kgf/cm^2^), a unit commonly used and accepted in clinical and veterinary pain studies. Although newtons per square centimeter (N/cm^2^) is the SI unit of force, kgf/cm^2^ remains standard in the pressure algometry literature.

All measurements were performed by M.C.-B., while PAS recorded the corresponding data in each horse’s individual study record. The average of the three consecutive PPT readings was used for analysis to determine variability.

**Figure 2:**
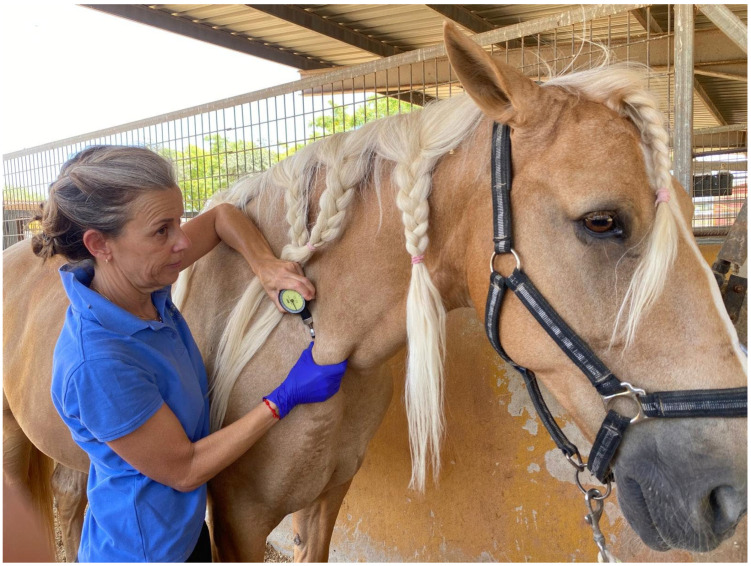
Maria Calatayud-Bonilla measuring pressure pain threshold with an algometer at the distal portion of the brachiocephalic muscle.

This algometry protocol was consistently applied at the following time points: (1) immediately prior to the intervention (baseline measurement); (2) immediately following the dry needling (DN) procedure; (3) four hours post-treatment; (4) twenty-four hours post-treatment; and (5) seventy-two hours post-treatment (Figure 2). To reduce circadian variability, all algometry measurements were performed in the morning [24].

### 2.4. Dry Needling (DN) Procedure

**Figure 3:**
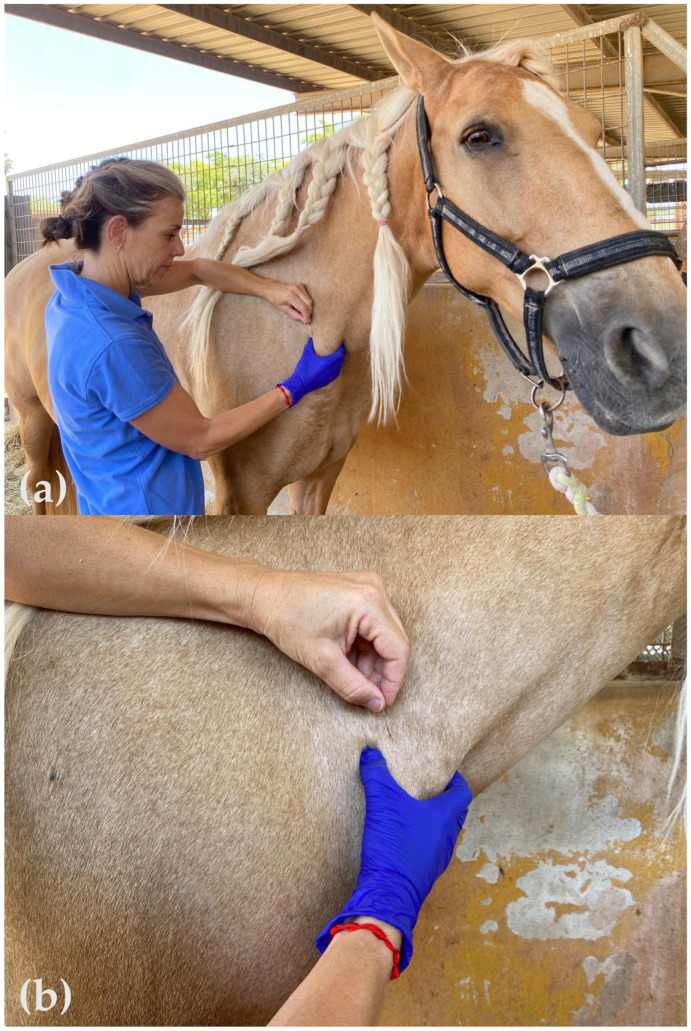
Dry needling procedure performed by Maria Calatayud-Bonilla. (**a**) Trigger point (TrP) in the distal brachiocephalic muscle identified via pincer palpation. (**b**) The needle was swiftly inserted through the skin, the guide tube removed, and the “Fast-in Fast-out” technique was applied to stimulate the TrP and elicit local twitch responses.

Following baseline PPT measurement, the physiotherapist performed the DN intervention according to a standardized and validated protocol, which was strictly adhered to throughout the study period. The procedure consisted of the following steps: (1) The most reactive TrP in the distal portion of the BM was identified via pincer palpation. (2) A sterile needle, mounted on a guide tube, was positioned directly above the identified TrP. (3) The needle was swiftly inserted through the skin, after which the guide tube was removed. (4) The “Fast-in Fast-out” technique [20,47,48,49,50] was applied to stimulate the TrP and elicit LTRs. (5) The number of LTRs was counted by the physiotherapist during a standardized 1 min stimulation period, with PAS recording the results. (6) After needle withdrawal, hemostasis was applied if necessary, and all relevant data were documented accordingly (Figure 3a,b).

### 2.5. Trigger Point Criteria (Palpation)

TrPs were defined as hypersensitive nodules within TBs in the distal portion of the BM, associated with a JS upon palpation. The cranial angle of the scapula was used as a consistent anatomical reference for TrP localization. The presence of at least one confirmed TrP with a JS was an inclusion criterion and was not subjected to inferential analysis.

### 2.6. Statistical Analysis

All statistical analyses were conducted using Generalized Linear Mixed Models (GLMMs) to appropriately handle the repeated-measures design and to model both continuous and categorical outcome variables. Continuous quantitative outcomes, namely PPTs, were analyzed using GLMMs assuming a Gaussian distribution with an identity link function. Categorical and count outcomes, including NRS, FTTS, and LTRs, were modeled using GLMMs with a Poisson distribution and log link function, suitable for modeling non-normally distributed count data. EPF scores and JS presence were evaluated assuming a binomial family with a logit link function.

All models for PPT analyses included treatment group (TG vs. CG), time (pre-treatment or post-treatment: 0 h, 4 h, 24 h, 72 h), body side (left/right), and housing condition (stabled vs. pastured) as fixed effects. Models for NRS and FTTS included time, housing condition, sex, age, and size as fixed effects. Models for LTRs included the aforementioned variables plus body side. Interaction terms between treatment group and time were included to evaluate the differential effect of the intervention across time points. Horse ID was included as a random intercept in all models to account for intra-subject variability.

We did not perform conventional univariate analyses (e.g., *t*-tests or chi-square tests) as a preliminary step. Instead, we adopted a hierarchical modeling strategy within the GLMM framework. A series of exploratory models were constructed to evaluate the isolated effects of each fixed factor. Only those factors showing significant main effects were considered in subsequent models evaluating two-way interactions, particularly the treatment group × time interaction, which was retained based on observed improvement in model fit and parsimony as indicated by Akaike’s Information Criterion (AIC).

Model selection and goodness-of-fit were assessed using AIC values, with lower AIC values indicating a better trade-off between model complexity and fit. Model validation included inspection of residual normality (for Gaussian models), evaluation of dispersion parameters (for Poisson models), and verification of random effect variance components to ensure appropriate modeling of repeated measures.

When statistically significant main effects or interactions were detected, Tukey’s HSD post hoc tests were performed to control for multiple comparisons. Statistical significance was set at *p* ≤ 0.05 for all analyses. All statistical analyses were performed using JASP software (version 0.18.3, University of Amsterdam, Amsterdam, The Netherlands).

The statistical power of the study was determined a posteriori using the online GLIMMPSE software tool (https://glimmpse.samplesizeshop.org, accessed on 23 March 2024), with alpha set at 0.05 and beta at 0.20 (power = 80%). Based on the sample size and observed variability, this study achieved an estimated power of 90% or higher to detect clinically meaningful differences in the primary outcome variables.

## 3. Results

In total, 98 horses participated in the study, with 66 assigned to the treatment group (TG) and 32 to the control group (CG). Most horses were geldings (CG: 75%; TG: 63.6%). Regarding age distribution, the majority of CG horses were between 5 and 10 years old (46.9%) or were older than 10 years (43.8%), with only 9.4% younger than 5 years. In the TG, 69.7% of horses were over 10 years, 27.3% were between 5 and 10 years, and 3.0% were younger than 5 years. Regarding bodyweight, most horses weighed over 500 kg (CG: 87.5%; TG: 63.6%), with 31.8% of TG horses weighing between 300 and 500 kg, and 4.5% under 300 kg.

In terms of aptitude, 68.8% of CG horses were categorized as competition horses (classical dressage) and 31.3% as non-sport horses. In the TG, 51.5% were competition horses (classical dressage), 28.8% were school horses, and 19.7% were non-sport horses. Housing conditions also varied: 87.5% of CG horses were fully stabled, while in the TG, 57.6% were stabled, 28.8% were kept in paddocks, and 13.6% were housed in mixed conditions.

During the study, nine horses were excluded: four geldings, three stallions, and two mares. Of these, three (two geldings and one stallion) were removed due to the development of problematic behavior.

All horses included in the final analysis exhibited at least one TrP in the BM and a clear JS during palpation. Although the total number of TrPs per horse was not recorded, the most sensitive TrP within the TB was consistently selected as the target for therapeutic intervention.

### 3.1. Algometry Results

**Figure 4:**
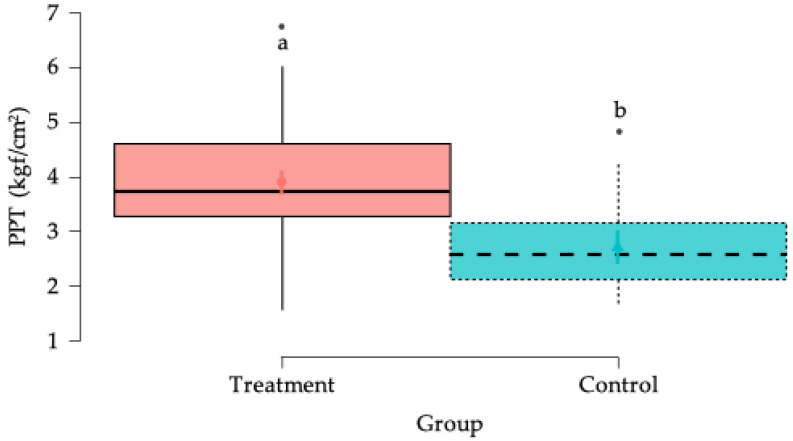
Box plots showing the estimated global mean values and 95% confidence intervals for algometry measurements (pressure pain threshold (PPT) kilogram-force/cm^2^ (kgf/cm^2^)) in the horses in the study according to the treatment factor. ^a,b^ = Groups with different lower-case letters denote significant differences (*p* < 0.001) measured using the Tukey test.

PPT values, expressed in kilogram-force per square centimeter (kgf/cm^2^), were analyzed using Generalized Linear Mixed Models (GLMMs). The initial model assessed the effects of treatment, body side, and their interaction. A statistically significant main effect of treatment was found (Figure 4), while neither the effect of body side (*p* = 0.599) nor the interaction between the treatment and side (*p* = 0.986) reached statistical significance (Table 1).

A second model including treatment and housing condition revealed a significant effect of treatment (*p* < 0.001), but no significant effects of housing condition (*p* = 0.459) or its interaction with treatment (*p* = 0.637) (Table 2).

Given that treatment was the only factor significantly influencing PPT values, it was subsequently modeled together with time, subdivided by week and hour, including the three-way interaction between treatment, week, and hour. The final model showed significant main effects of treatment (*p* < 0.001), week (*p* < 0.001), and hour (*p* < 0.001), as well as a significant three-way interaction between treatment, week, and hour (*p* < 0.001) (Table 3).

Overall, PPT values were significantly higher in the TG compared to the CG. In both groups, PPT values were significantly affected by time. However, the most important finding was the significant interaction between treatment and time (week and hour), demonstrating a clear improvement in PPT responses over time in the TG compared to the CG.

In general, PPT values in the CG remained relatively stable across the study period, while in the TG, PPT values progressively increased, especially after the second week of treatment, with significant differences observed at all post-treatment time points (1 h, 4 h, 24 h, and 72 h) during weeks 2 and 3.

**Figure 5:**
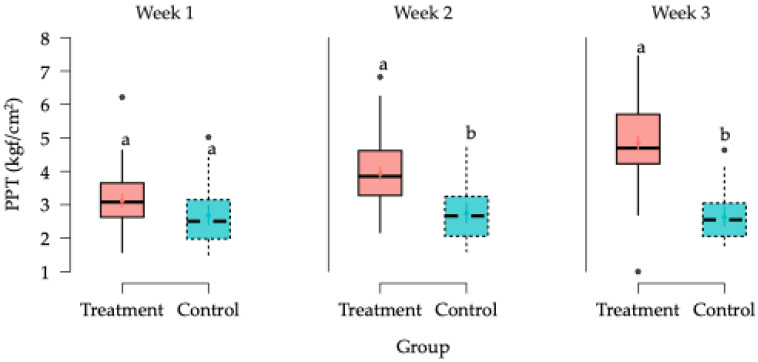
Box plots showing the estimated global mean values and 95% confidence intervals for algometry measurements (PPT) (kgf/cm^2^)) in the horses in the study according to the treatment per week factors. ^a,b^ = Groups with different lower-case letters denote significant differences (*p* < 0.001) measured using the Tukey test.

**Figure 6:**
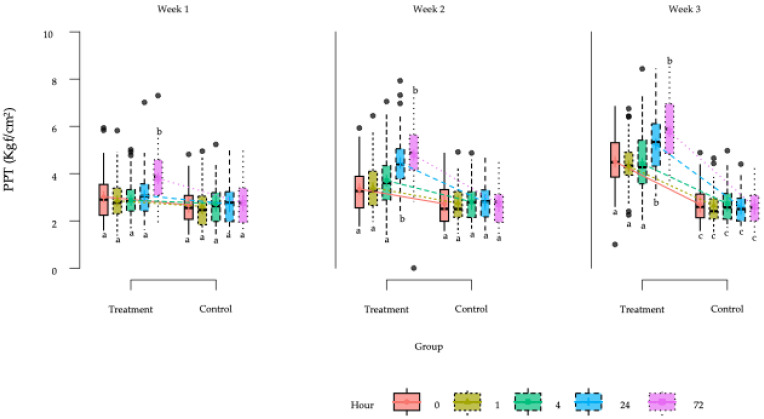
Box plots showing the estimated global mean values and 95% confidence intervals for algometry measurements (PPT) (kgf/cm^2^)) in the horses in the study according to the treatment per week per hour factors. ^a–c^ = Groups with different lower-case letters denote significant differences (*p* < 0.001) measured using the Tukey test.

During the first week, no statistically significant differences between groups were observed at 0 h, 1 h, 4 h, or 24 h; however, a significant difference emerged at 72 h. In weeks 2 and 3, significant differences were present at all evaluated time points, with markedly higher PPT values in the TG. The largest effect sizes were observed during week 3. Estimated marginal means for PPTs across treatment groups, weeks, and time points are presented in Figure 5 and Figure 6.

### 3.2. Results from Categorical and Count Outcomes

#### 3.2.1. Functional Total Test Score

The FTTS was derived from responses to a functional questionnaire consisting of seven questions regarding the BM’s function, completed by the owners before each treatment session. In the model assessing FTTS, a significant (*p* < 0.001) effect was found when comparing the horses’ progress over the weeks (Table 4).

**Figure 7:**
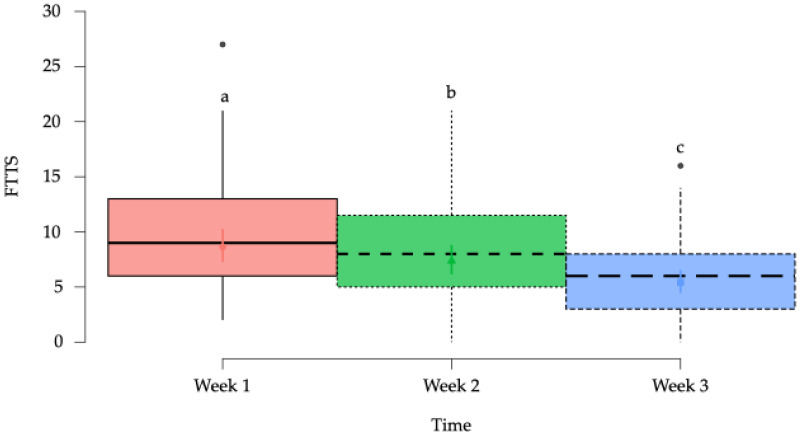
Box plots showing the estimated mean values and 95% confidence intervals for the functional test total score (FTTS) in the horses in the study according to time. ^a–c^ = Groups with different lower-case letters denote significant differences (*p* < 0.001) measured using the Tukey test.

The estimated marginal means showed a significant a progressive decrease over the three-week period. Statistically significant differences were observed between weeks 1, 2, and 3 (Figure 7).

#### 3.2.2. Numerical Rating Scale

The NRS is a subjective measure where owners rated their horses’ pain on an 11-point scale from 0 to 10. This study evaluated whether owners’ pain assessments correlated with algometry measurements. A significant effect was found in the progression of owners’ reported pain before each treatment session (*p* < 0.001); however, the rest of the fixed factors did not affect the model (Table 5).

**Figure 8:**
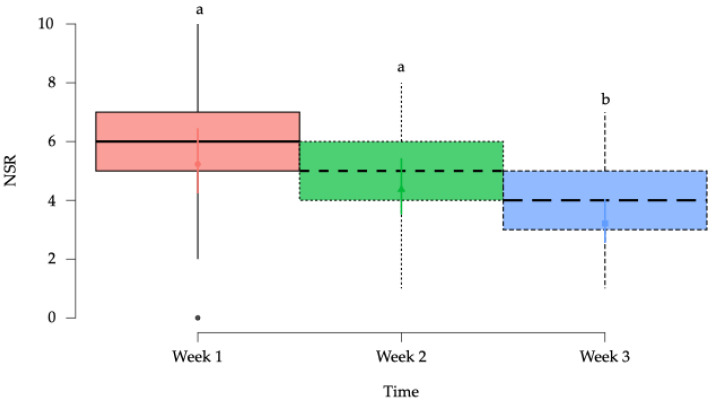
Box plots showing the estimated mean values and 95% confidence intervals for numerical rating scales (NRS) in the horses in the study according to the time factor. ^a,b^ = Groups with different lower-case letters denote significant differences (*p* < 0.001) measured using the Tukey test.

NRS showed similar values at week 1 and 2; however, the values for this variable were significantly *p* < 0.001 lower at week 3 when compared to weeks 1 and 2 (Figure 8).

#### 3.2.3. Local Twitch Responses (LTRs)

The number of LTRs was recorded bilaterally across the three treatment sessions to evaluate their correlation with pain progression. LTRs were significantly affected by time and body side (either right or left) in the exploratory model. The rest of the fixed factors did not influence the model (Table 6). On the other hand, there was not a significant effect when the interaction between time and body side was evaluated (*p* = 0.064).

**Figure 9:**
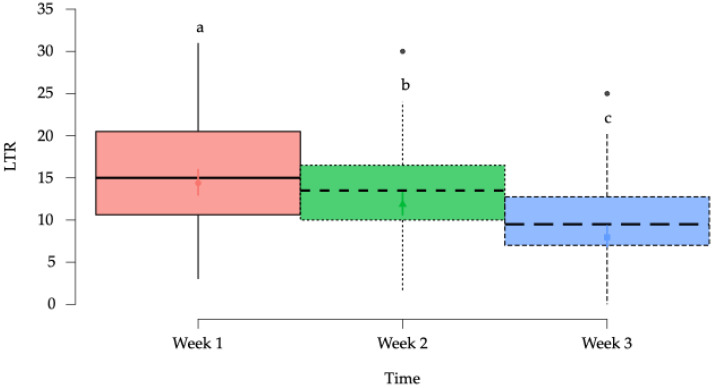
Box plots showing the estimated mean values and 95% confidence intervals for number of local twitch responses (LTRs) according to time factor. ^a–c^ = Groups with different lower-case letters denote significant differences (*p* < 0.001) measured using the Tukey test.

**Figure 10:**
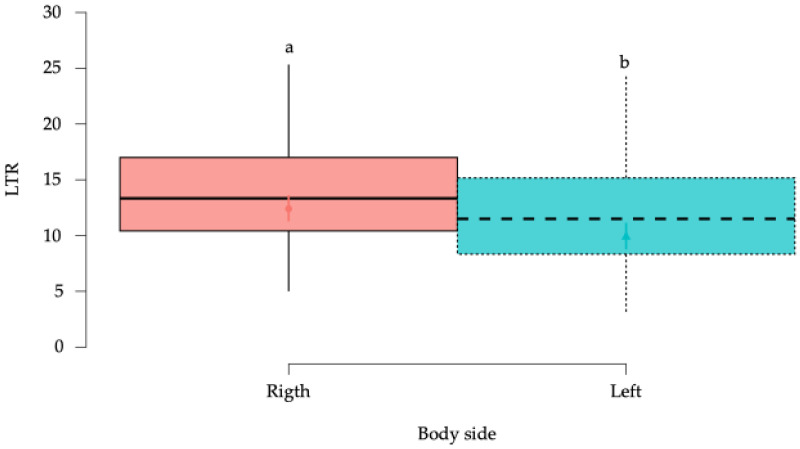
Box plots showing the estimated mean values and 95% confidence intervals for number of LTRs according to the body side factor. ^a,b^ = Groups with different lower-case letters denote significant differences (*p* < 0.001) measured using the Tukey test.

A statistically significant reduction week-by-week in LTRs over time was observed in the horses of the study (*p* < 0.001) (Figure 9), with a significant (*p* < 0.001) reduction in the LTRs in the left side when compared to the right side (Figure 10).

#### 3.2.4. Equine Pain Face

The EPF was assessed for its clinical value in detecting myofascial pain related to TrPs in the BM. However, this variable was not affected by the fixed factors evaluated in the model (Table 7).

#### 3.2.5. Jump Sign (JS)

JS registers (presence or absence) were significantly (*p* = 0.002) affected time factors. The rest of the fixed factors did not influence in the model (Table 8).

Estimated marginal means indicated a slight decrease in probability at Time 3 (EMM = 0.953, 95% CI [0.212, 0.999]). However, post hoc pairwise comparisons using Tukey adjustment revealed no significant differences between individual time points (all *p* > 0.95).

## 4. Discussion

This study assessed the effectiveness of DN in relieving myofascial pain associated with TrPs in the BM, both immediately (≤72 h) and in the short term (1–3 weeks). PPT significantly improved at 72 h, with the most pronounced effect observed after three DN sessions. These findings support DN’s effectiveness in reducing pain at 72 h and three weeks, consistent with human studies [26,50,51,52,53,54,55,56]. This is the first study to examine DN’s efficacy for equine myofascial local pain, following a preliminary report [41].

The physiotherapist, blinded to outcome measures and assisted by PAS during data collection, performed all interventions following a standardized protocol. While the practitioner had over a decade of experience in TrP therapy [57], no formal intra-assessor repeatability testing was conducted. Therefore, we acknowledge that the findings may have been influenced, in part, by individual practitioner technique and skill, representing a potential source of bias inherent in manual therapeutic studies. A total of 98 horses were included, achieving 80% power at a 0.005 significance level. Horses were randomly assigned to groups.

Palpation, reliable [42] and clinically relevant for diagnosing equine musculoskeletal pain [24], was used to evaluate local pain. An algometer was chosen for its reproducibility, user-friendliness [58], clinical utility [59], and reliability [60]. PPT was assessed before treatment, immediately after, and at 4, 24, and 72 h post-treatment [59]. Three consecutive measurements were taken at each time point [24,45,58,61,62,63], with 4 s intervals [59], shorter than those used in most human studies [57,63,64].

Maintaining a constant rate of pressure during the 5 s algometry testing window is considered crucial [58,59], and achieving this was technically challenging in live horses under field conditions, as previously noted [24]. Even when pressure is applied steadily, minor variations in rate and examiner control are likely and represent a known limitation in this type of nociceptive threshold assessment.

Nine horses were excluded, including three due to behavioral issues [24]. The frequency of measurements and potential discomfort [24,45] may have affected sensitivity, possibly due to “wind-up” phenomena [65]. Although the algometer can be perceived as aversive [24], this was not observed in the remaining horses.

Palpating TrPs induces pain in horses [10,11,21,44]. Distinguishing active from latent TrPs is not possible in horses due to their inability to verbally report symptoms [31,33,35]. A previous study found higher TrP-related pain in sport horses (*p* < 0.0001) [42], but this study found no significant influence of habitat (*p* = 0.637) or fitness (*p* = 0.867) on treatment outcomes. Although human studies suggest that physical activity affects pain perception, its effect on PPT remains unclear [39,66]. High-intensity activity may reduce PPT, while low-intensity activity may decrease sensitivity [67], indicating a complex relationship between exercise, pain, and treatment [39].

Post-treatment, horses were restricted from exercise for 72 h to minimize BM activation. In humans, controlled exercise can restore PPT in 24 h [61], and low-load exercise may reduce PNS [68,69]. In this study, improvements occurred despite exercise restriction. Future research could evaluate whether BM activation accelerates recovery and shortens PNS duration in horses, as seen in humans [14,68,69].

The necessity of eliciting LTRs remains debated [14]. Previously considered essential for TrP deactivation [20,49,70], more recent studies challenge this view [71,72]. This study aimed to elicit LTRs based on their role in reducing substance *p* [49,73,74] and improving treatment efficacy [75]. A progressive reduction in LTRs was associated with pain reduction [40]. The intervention, applied once weekly for one minute, respected muscle regeneration times [72]. DN was performed using the “fast-in, fast-out” technique [70], which has shown high effectiveness in pain reduction [76].

PNS is a common side effect of DN [48,72]. While higher DN doses may relieve pain, they also increase the risk of hemorrhage, tissue damage, and inflammation [48,72]. Although EMG alterations have been observed after high doses [49], no direct relationship has been found between dose, PNS, and LTRs [48,77]. In fact, more LTRs may enhance therapeutic effect without increasing PNS [64]. To minimize PNS, needling time was limited, and only one needle was used per TrP, allowing for unrestricted LTRs.

Several studies indicate that PNS resolves spontaneously within 36–48 h [50,57,77]. In this study, algometry values decreased after DN—suggesting increased pain—peaked at 24 h, and normalized by 72 h, in line with prior reports [48,50].

It remains uncertain whether horses experience PNS, given their inability to verbally report symptoms. In humans, tools such as PPT and VAS help distinguish PNS from TrP-related pain [61]. Lacking equivalent tools for horses, the post-DN decrease in PPT could not be definitively attributed to PNS. However, higher algometry values at 72 h compared to baseline suggest overall pain improvement after this period.

Although facial expressions and JS are considered reliable indicators of equine pain, no significant changes were observed throughout the intervention. The EPF may not detect TrP-induced discomfort, perhaps due to the low-intensity stimulus compared to acute nociceptive triggers. Further studies are needed to evaluate its sensitivity to myofascial pain. Notably, despite reduced TrP pain, some horses continued to show JS without a clear clinical explanation.

Horse owners reported significant improvements in their animals’ condition. NRS scores showed progressive reduction in perceived pain across the intervention, consistent with previous studies [50,57,63], confirming the validity of owner-reported outcomes.

The BM is essential for cervical motion and forelimb protraction [78], functions directly related to athletic performance in horses. Pain or dysfunction in this muscle alters the cranial phase of the stride and reduces contralateral lateroflexion [21], both of which were indirectly evaluated through the Functional Total Test Score (FTTS). This observational tool, based on owner-reported behaviors such as rein contact, lateral flexion, and forelimb engagement, showed significant improvements following DN. These findings support the potential role of DN not only in alleviating local pain, but also in enhancing functional capacity. Although the FTTS has not yet undergone formal validation, its consistency with objective measures (i.e., algometry, NRS) and focus on performance-related behaviors suggest that it may be a promising instrument for evaluating musculoskeletal function in clinical and research settings. Further studies are needed to validate its structure and assess its applicability across other muscle groups and equine disciplines.

A key limitation of this study is its exclusive focus on the BM. While the results support DN’s efficacy in this region, they cannot be generalized to other anatomical areas affected by myofascial pain. Future studies should include additional muscle groups to capture a more holistic understanding of myofascial dysfunction in horses and evaluate the systemic impact of DN across the musculoskeletal system.

Another limitation was the lack of standardized measurement of needle insertion depth during DN. Although the intervention was performed by a qualified physiotherapist with anatomical precision, depth was not quantified or recorded. As needle depth may influence both elicitation of LTRs and therapeutic effect, future studies should consider objectively documenting this variable to improve reproducibility and control for its potential impact.

To ensure that the benefits of DN for equine myofascial pain reach a broader audience, dissemination strategies should include social media platforms such as Instagram and Twitter. A recent study [79] demonstrated that Instagram can effectively communicate complex veterinary topics. Transparent and engaging communication fosters public trust, counteracts misinformation, and enhances the societal impact of equine research.

## 5. Conclusions

This study demonstrates that DN is effective in reducing local pain elicited by palpation of TrPs in the BM, both immediately (within 72 h) and in the short term (over three weeks). The progressive pain reduction correlated with a decrease in LTRs. PNS peaked at 24 h post-treatment and resolved within 72 h. Although the benefits of restricting exercise after DN are not yet well established, the time course of PNS suggests that limiting the activity of the treated muscle during the first 72 h may be advantageous.

Despite the improvement in local pain, horses continued to display the JS, indicating that this reaction may not be directly associated with pain. The EPF was not sensitive enough to detect TrP-induced pain in the BM. The NRS effectively reflected the reduction in local pain from the owners’ perspective, and the FTTS appears to be a useful tool for evaluating muscle function.

Dry needling appears to be a promising and effective non-pharmacological intervention to reduce local muscle pain in horses, potentially enhancing their comfort, movement, and performance.

## Figures and Tables

**Table 1:** Generalized Linear Mixed Model (GLMM) of average algometry value according to the fixed factors and their interaction.

Effect	df	ChiSq	*p* Value
Intercept	1	247.274	<0.001
Treatment	1	31.916	<0.001
Side	1	0.277	0.599
Treatment × Side	1	2.906 × 10^−4^	0.986

df: Degrees of freedom.

**Table 2:** GLMM on average algometry value according to the fixed factors.

Effect	df	ChiSq	*p* Value
Intercept	1	164.543	<0.001
Group	1	14.636	<0.001
Housing	2	1.558	0.459

df: Degrees of freedom.

**Table 3:** GLMM on average algometry value according to the fixed factors and their interaction.

Effect	df	ChiSq	*p* Value
Intercept	1	256.813	<0.001
Group	1	36.711	<0.001
Week	2	68.558	<0.001
Hour	4	103.919	<0.001
Group × Week × Hour	8	54.411	<0.001

df: Degrees of freedom.

**Table 4:** GLMM on functional test total score (FTTS) according to the fixed factors.

Effect	df	ChiSq	*p* Value
Intercept	1	50.490	<0.001
Time	2	44.707	<0.001
Sex	2	5.393	0.067
Age	2	0.391	0.822
Size	2	3.996	0.136
Housing	2	3.845	0.146

df: Degrees of freedom.

**Table 5:** GLMM on the numerical rating scale (NRS) according to the fixed factors.

Effect	df	ChiSq	*p* Value
Intercept	1	79.591	<0.001
Time	2	30.478	<0.001
Sex	2	3.234	0.198
Age	2	6.457	0.060
Size	2	2.223	0.329
Housing	2	4.876	0.087

df: Degrees of freedom.

**Table 6:** GLMM on local twitch responses (LTRs) according to the fixed factors.

Effect	df	ChiSq	*p* Value
Intercept	1	142.997	<0.001
Time	2	33.714	<0.001
Body side	1	14.743	<0.001
Sex	2	2.519	0.284
Age	2	3.328	0.189
Size	2	2.870	0.238
Housing	2	1.385	0.500

df: Degrees of freedom.

**Table 7:** GLMM on equine pain face (EPF) according to the fixed factors.

Effect	df	ChiSq	*p* Value
Intercept	1	10.909	<0.001
Time	2	4.891	0.087
Sex	2	2.014	0.365
Age	2	1.061	0.588
Size	2	3.373	0.185
Housing	2	0.651	0.722

df: Degrees of freedom.

**Table 8:** GLMM on jumping sign (JS) presence according to the fixed factors.

Effect	df	ChiSq	*p* Value
Intercept	1	16.795	<0.001
Time	2	12.665	0.002
Body side	1	0.331	0.109
Sex	2	4.436	0.365
Age	2	4860	0.088
Size	2	0.000	1.000
Housing	2	4.569	0.102

df: Degrees of freedom.

## Data Availability

The data presented in this study are available on request from the corresponding author. The data are not publicly available due to privacy and ethical restrictions.

## Abbreviations

The following abbreviations are used in this manuscript:

MPS	Myofascial Pain Syndrome
TrPs	Trigger Points
DN	Dry Needling
TB	Taut Band
LTR	Local Twitch Response
SEA	Spontaneous Electrical Activity
JS	Jump Sign
NRS	Numerical Rating Scale
VAS	Visual Analogue Scale
PPT	Pressure Pain Threshold
PNS	Post Needling Soreness
BM	Brachiocephalic Muscle
FTTS	Functional Total Test Score
TG	Treatment Group
CG	Control Group
PI	Principal Investigator
AS	Assistant
EPF	Equine Pain Face

## Appendix A

Functional questionnaire (FTTS):

1. Does your horse tend to brace against the bit?
2. Does your horse show increased resistance or heaviness on the left rein?
3. Does your horse have difficulty performing lateral movements (e.g., leg-yield, shoulder-in)?
4. Does your horse struggle to laterally bend the cervical spine?
5. Does your horse show limited ability to flex the cervical spine (longitudinal flexion)?
6. Have you observed a reduction in forelimb protraction or stride length?
